# Natural Function and Structural Modification of Climacostol, a Ciliate Secondary Metabolite

**DOI:** 10.3390/microorganisms8060809

**Published:** 2020-05-27

**Authors:** Federico Buonanno, Elisabetta Catalani, Davide Cervia, Cristina Cimarelli, Enrico Marcantoni, Claudio Ortenzi

**Affiliations:** 1 Laboratory of Protistology and Biology Education, Department of Education, Cultural Heritage, and Tourism (ECHT), Università degli Studi di Macerata, 62100 Macerata, Italy; federico.buonanno@unimc.it; 2 Department for Innovation in Biological, Agro-food and Forest systems (DIBAF), Università degli Studi della Tuscia, 01100 Viterbo, Italy; ecatalani@unitus.it (E.C.); d.cervia@unitus.it (D.C.); 3 School of Science and Technology, Section of Chemistry, Università degli Studi di Camerino, 62032 Camerino, Italy; cristina.cimarelli@unicam.it (C.C.); enrico.marcantoni@unicam.it (E.M.)

**Keywords:** *Climacostomum virens*, secondary metabolites, predator–prey interactions, resorcinolic lipids, ciliates, natural products

## Abstract

The review highlights the main results of two decades of research on climacostol (5-[(2*Z*)-non-2-en-1-yl]benzene-1,3-diol), the resorcinolic lipid produced and used by the ciliated protozoan *Climacostomum virens* for chemical defense against a wide range of predators, and to assist its carnivorous feeding. After the first studies on the physiological function of climacostol, the compound and some analogues were chemically synthesized, thus allowing us to explore both its effect on different prokaryotic and eukaryotic biological systems, and the role of its relevant structural traits. In particular, the results obtained in the last 10 years indicate climacostol is an effective antimicrobial and anticancer agent, bringing new clues to the attempt to design and synthesize additional novel analogues that can increase or optimize its pharmacological properties.

## 1. Introduction

**Figure 1:**
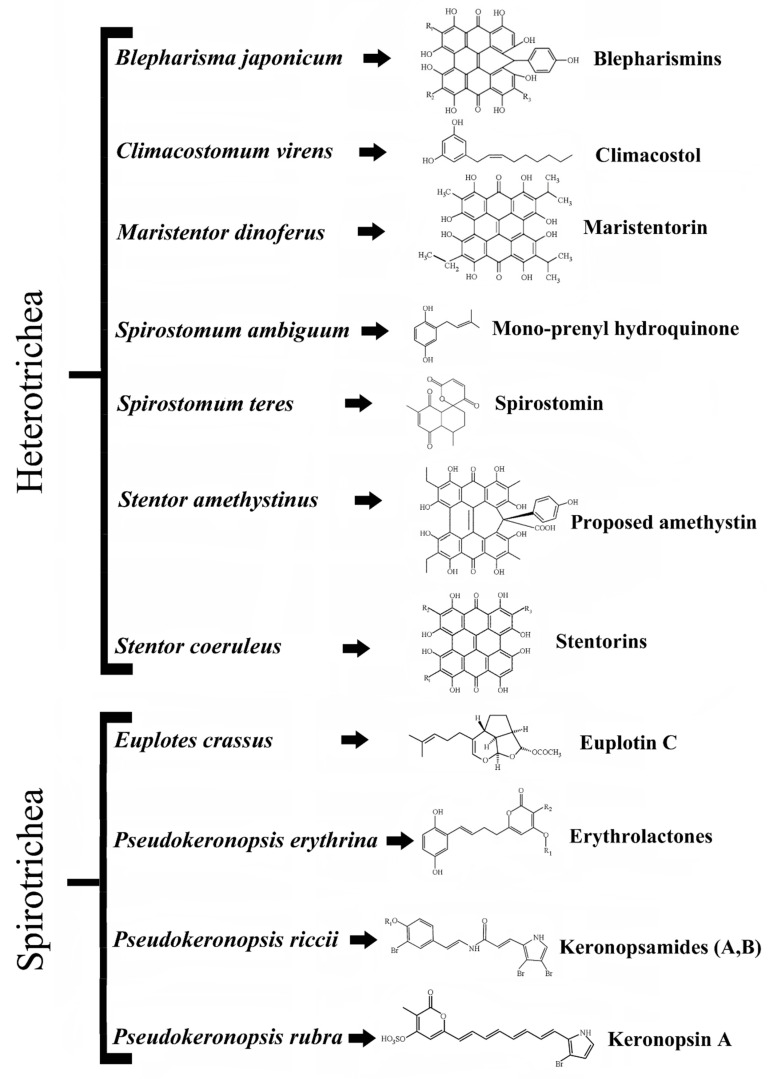
Relevant secondary metabolites with biological activities produced by ciliated protists. Blepharismins: BP-A (R_1_ = C_2_H_5_; R_2_ = C_2_H_5_; R_3_ = H); BP-B (R_1_ = C_2_H_5_; R_2_ = *i*-C_3_H_5_; R_3_ = H); BP-C (R_1_ = R_2_ = *i*-C_3_H_5_; R_3_ = H); BP-D (R_1_ = C_2_H_5_; R_2_ = *i*-C_3_H_5_; R_3_ = CH_3_); BP-E (R_1_ = R_2_ = *i*-C_3_H_5_; R_3_ = CH_3_). Stentorins: S-C (R_1_ = *i*-C_3_H_5_; R_2_ = *i*-C_3_H_5_; R_3_ = H). Erythrolactones: E-1 (R_1_ =SO_3_; R_2_ = C_6_H_13_); E-2 (R_1_ =SO_3_; R_2_ = C_7_H_15_); E-3 (R_1_ =SO_3_; R_2_ = C_8_H_17_); E-4 (R_1_ =H; R_2_ = C_6_H_13_); E-5 (R_1_ =H; R_2_ = C_7_H_15_); E-6 (R_1_ =H; R_2_ = C_8_H_17_). Redrafted from [3] and reproduced under the Creative Commons attribution 3.0 license: https://creativecommons.org/licenses/by/3.0/.

Aquatic organisms live in environments which are exceptionally full of competitive biodiversity and for this reason have evolved a great number of adaptive strategies to survive and reproduce, which in eukaryotic unicellular microbes (protists) appear particularly well represented. At the molecular level, ciliated protists show adaptive strategies based on at least two classes of compounds, pheromones and secondary metabolites. Pheromones are diffusible proteins produced and secreted by different ciliate species to mediate self–nonself recognition mechanisms, responsible for cell shift between the vegetative and sexual stages of the cell biological cycle (see [1] for a review). Secondary metabolites produced by ciliates include toxic substances either stored in the cell cytoplasm or inside ejectable organelles (known as extrusomes) that are anchored to the cell cortex before being used in predator-prey and cell-environment interactions [2,3]. These secondary metabolites are synthesized through different biogenetic routes and have been named from the taxonomic names of their source organisms. Some metabolites are biosynthesized via the polyketide pathway (or mixed biogenetic routes), whilst several others derive from the classical terpenoid biogenesis (see [3,4] for a review). Representative examples are the terpenoid euplotins from *Euplotes* species [5] and some polycyclic aromatic compounds built on hypericin structure such as blepharismins from *Blepharisma* species [6,7], stentorins from *Stentor coeruleus* [8], amethystin from *Stentor amethystinus* [9], and maristentorin from *Maristentor dinoferus* [10]. Other toxic secondary metabolites are spirostomin from *Spirostomum teres* [11] and mono-prenyl hydroquinone from *Spirostomum ambiguum* [12]. Keronopsins, keronopsamides and erythrolactones are, instead, produced by three different species of the genus *Pseudokeronopsis*: *P. rubra*, *P. riccii* and *P. erythrina* [7,13,14,15,16], respectively (Figure 1).

**Figure 2:**
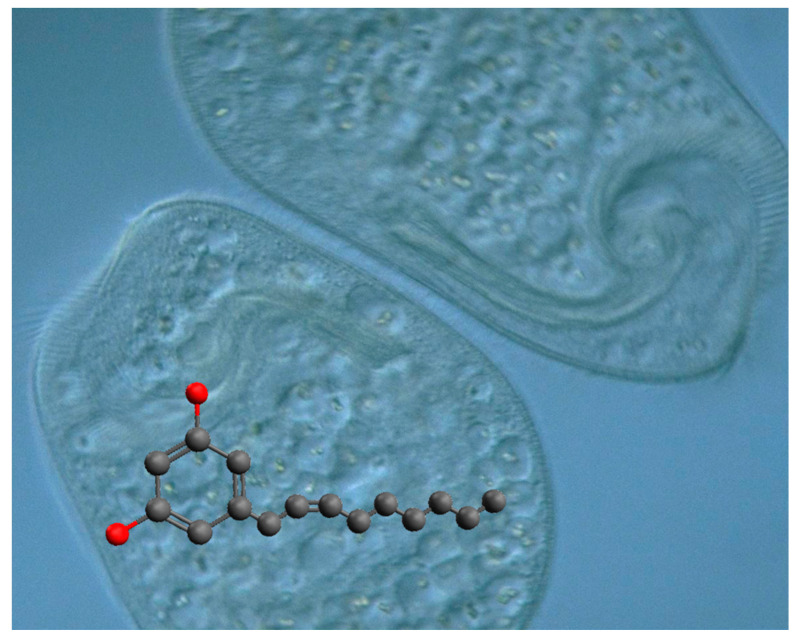
*Climacostomum virens* and molecular structure of its secondary metabolite, climacostol. Original microphotograph of *C. virens* by Y. Tsukii (Protist Information Server, http://protist.i.hosei.ac.jp/Protist_menuE.html).

Climacostol (5-[(2*Z*)-non-2-en-1-yl]benzene-1,3-diol) is a toxic secondary metabolite physiologically produced by the freshwater ciliate *Climacostomum virens* (Figure 2) for chemical defense against unicellular and multicellular predators, or for chemical offence to assist its carnivorous feeding [3,17]. This molecule is formed by a phenolic skeleton and a long unsaturated aliphatic hydrocarbon chain attached to the ring structure, and belongs to resorcinolic lipids (or alkenylresorcinols), a group of natural amphiphilic compounds detected in both prokaryotes and eukaryotes. The 5-alkenylresorcinols usually have an isolated double bond in the chain portion of the molecule, while examples with a conjugated double bond with aromatic ring are unusual [18]. A possible pathway for the biosynthesis of climacostol was proposed from the C16-polyketide, with a cyclization and a decarboxylation [19], and the toxin was also obtained by chemical synthesis [19,20]. More recently, climacostol has been synthetized as a pure compound in the natural and most bioactive *Z*-configuration by a novel and straightforward synthesis [21], which has allowed researchers to better study its effects on different biological systems.

## 2. Climacostol Mediates Predator–Prey Interactions

*Climacostomum virens* is a noncontractile freshwater heterotrich ciliate, which often looks green due to the presence of endosymbiotic algae (*Chlorella* sp.). The green cells can be cultivated in the dark to reduce or eliminate the symbionts, in order to obtain colorless strains [17,22]. *C. virens* possess numerous extrusomes reported as cortical granules (or granulocysts) which are able to discharge their content to the outside of the cell in response to external stimuli. The colorless extruded substances are mainly represented by climacostol with the presence of some related analogues [16,18]. The defensive function of climacostol was first demonstrated against the raptorial ciliate *Dileptus margaritifer*. Indeed, when this predator touches a cell of *C. virens* with its toxicyst-bearing proboscis, *D. margaritifer* swims backward and a condensed substance, raised from the site of contact, becomes visible under a dark-field microscope. After other attacks by the same individual, the proboscis of *D. margaritifer* appears shorter and visibly damaged, whereas subsequent attacks can lead to the death of the predator due to the cytotoxic effect of climacostol [17]. More recently, the toxic action of climacostol against metazoan predators was also demonstrated [23], and it was furthermore assumed that *C. virens* is able to use climacostol for chemical offense, to paralyze and kill prey [3].

## 3. Climacostol Exerts Cytotoxic and Antimicrobial Effect

**Figure 3:**
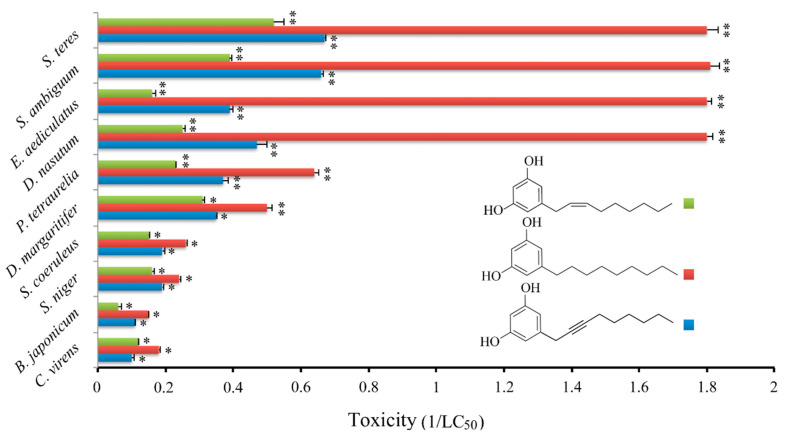
Cytotoxic effect of climacostol and its alkyl and alkynyl derivatives on ten ciliate species. Cell viability was assessed after 24 h, and each bar represents the mean (±SE) of 3 independent experiments. * *p* < 0.05 and ** *p* < 0.001. Original data from [24].

Initially, the cytotoxic effect of climacostol was analyzed against a panel of nine free-living ciliate species which share the same microhabitat with *C. virens*: *Blepharisma japonicum*, *Didinium nasutum*, *Dileptus margaritifer*, *Euplotes aediculatus*, *Paramecium tetraurelia*, *Spirostomum ambiguum*, *Spirostomum teres*, *Stentor coeruleus*, and *Stentor niger*. It was demonstrated that the cytotoxic potency of climacostol is related to the molecular structure of the unsaturated side chain, and that it could be modulated by the substitution of the double bond with a single or a triple one at the C_8_ position [24]. In fact, while the saturated alkyl derivative showed the highest cytotoxicity against the tested species, the alkynyl derivative that carries a triple bond in the side chain showed the lowest cytotoxic potency. An intermediate cytotoxicity was revealed by the native climacostol (with a double bond in the side chain) (Figure 3).

In a further study, the effect of climacostol and its alkyl and alkynyl derivatives was also tested on some Gram-positive and Gram-negative pathogen bacteria and on the fungus *Candida albicans* [25]. The results showed an appreciable and comparable antimicrobial activity of the three compounds, which were effective against Gram-positive bacteria and *C. albicans* with Minimum Inhibitory Concentrations (MIC) and Minimum Bactericidal Concentration (MBC) ranging from 8 to 32 mg L^−1^. By contrast, no significant toxicity against Gram-negative species (*Escherichia coli* and *Pseudomonas aeruginosa*) has been observed [25]. The authors speculated that this limited effect observed on the Gram-negative species can be explained by the peculiar structure of the bacterial cell wall. The outer membrane is indeed a selective barrier to the penetration of several compounds, due to the hydrophilic nature of the surface exposed to the environment and to the selectivity of the outer membrane proteins. To summarize, the overall results suggest that the saturation rate of the side chain of climacostol does not couple to its antimicrobial activity, whereas it is closely related to the cytotoxic action against ciliated protists. Therefore, it is likely that the general structure of the two moieties of climacostol, i.e., the di-hydroxy-phenyl group and the alkenyl chains, contributes to the antibiotic action as a whole. In general, the presence of a double bond in the hydrocarbon chain is associated with an increase in cell viability as compared to the saturated compounds, and an increase in the chain length does not interfere with the effect of aromatic ring substituents, which is clearly predominant [26].

## 4. Climacostol Reduces Tumor Progression via p53-Dependent Apoptosis 

On the basis of anticancer activity displayed by a number of other resorcinolic lipids (see [27] for a review), the effects of climacostol were initially explored *in vitro* on some human and rodent tumor cell lines [28]. In particular, experiments performed on human tumor squamous carcinoma A431 cells, and human promyelocytic leukemia HL60 cells demonstrated that climacostol exerts its action by inhibiting cell growth and triggering a mitochondrion-dependent apoptotic program. Subsequent extensive *in vitro* screenings and *in vivo* experiments confirmed the previous observations [4,21,29,30,31]. Although climacostol cytotoxicity appears more selective against tumors than certain immortalized nontumor cells [21,29], recent data suggest that climacostol effects are not necessarily correlated to the cancerous origin of cells [31]. However, the possibility that climacostol preferentially affects cancerous vs. normal (nontransformed non-immortalized) cells requires further investigation.

With regard to the action mechanism of climacostol, it was demonstrated that the protozoan toxin can trigger apoptosis by binding to nuclear and mitochondrial DNA, and promoting their cleavage after the generation of reactive oxygen species (ROS) in the presence of Cu(II) [32,33]. It is known that the induction of DNA damage could be effective in treating cancer, and many currently employed antitumor drugs, for example, platinum agents, function by means of this mechanism [34,35,36]. In this role, it was shown that cisplatin and climacostol do not display any additive effects on melanoma viability [29].

**Figure 4:**
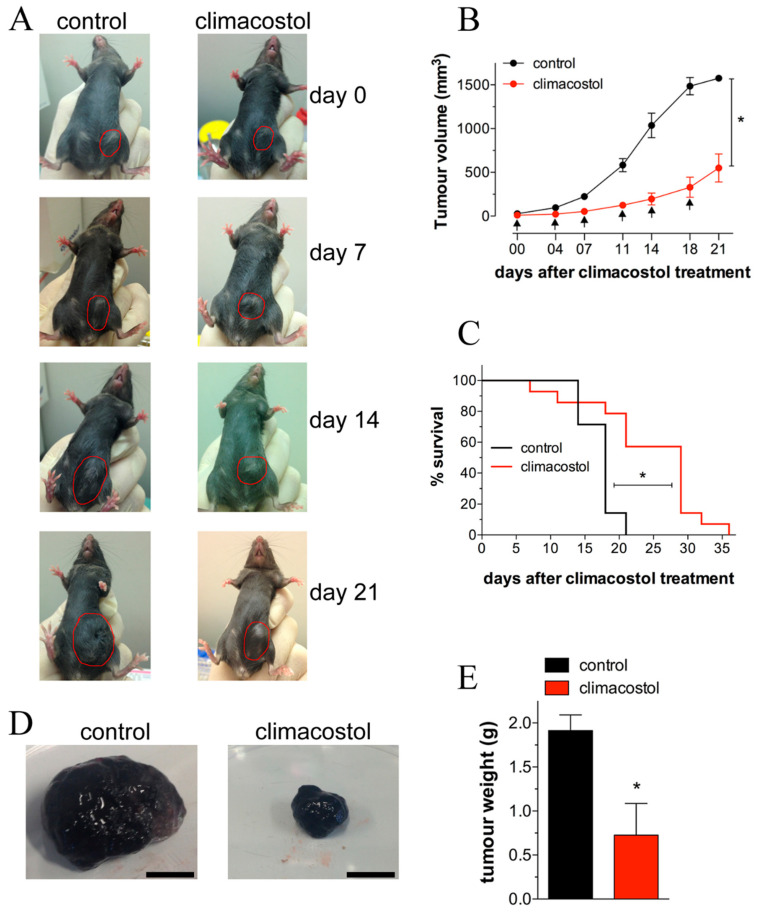
In vivo antitumor properties of climacostol in mice bearing melanoma allografts. When the B16-F10 syngeneic implantation was established, animals were intratumor injected with vehicle (control) or climacostol at 600 μg/mL every 3–4 days for 3 weeks. (**A**) Typical photographs taken at different time points and depicting the growth of subcutaneous melanomas, as indicated by red circles. (**B**) Tumor growth monitored by means of external caliper measurements and volume calculation. Arrows indicate the day of climacostol treatment. (**C**) Percentage survival analyzed by Kaplan–Meier curve. Images and data points in (**A**–**C**) represent the results obtained from 7–15 animals per experimental group. (**D**) Typical photographs of subcutaneous melanoma allografts excised from mice at day 16 of treatment (on day 0 and then every 3–4 days) with vehicle (control) or climacostol (600 μg/mL). Scale bar: 5 mm. (**E**) Weight of the excised tumors. Images and data in (**D**, **E**) represent the results obtained from 3 animals per experimental group. * *p* < 0.001 vs the respective control. Original picture from [29] and reproduced under the Creative Commons attribution 4.0 license: http://creativecommons.org/licenses/by/4.0/.

Recent investigations on the mechanisms of action of climacostol demonstrated that it reduces the viability/proliferation of melanoma cells, causing rapidly occurring DNA damage, also inducing the intrinsic apoptotic pathway characterized by the dissipation of the mitochondrial membrane potential, the translocation of Bax to the mitochondria, the release of cytochrome c from the mitochondria, and the activation of caspase 9-dependent cleavage of caspase 3 [29,30]. Additionally, persistent inhibition of the growth of melanoma allografts as well as a reduction in the number of viable and proliferating tumor cells was achieved after intratumoral injections of the toxin [4,29]. The notion that climacostol may induce a decrease in the microvessel sprouting that contributes to inhibition of melanoma growth was also suggested [4]. As shown in Figure 4, a significant improvement in survival of transplanted mice was reported, together with a decrease of tumor weight, and a reduction of viable cells inside the tumor. Of interest, the signaling events responsible for the climacostol-induced pro-apoptotic effects rely on the upregulation of the p53 network and its targets Noxa and Puma [4].

## 5. Climacostol Induces Dysfunctional Autophagy in Tumor Cells

In all the eukaryotic cells, a pivotal role for the maintenance of homeostasis is played by autophagy, the highly conserved process which operates via the degradation of cytoplasmic organelles, proteins, and macromolecules, and the recycling of the breakdown products. As autophagy supports cell survival or activates death pathways, it may represent a potential target for the available library of anticancer substances and for the development of new molecules.

Some of us recently reported on how climacostol regulates autophagy and the involvement of p53-dependent mechanisms [30]. Essentially, our data indicated that the protozoan toxin potently and selectively impairs autophagy in multiple tumor cells that are committed to dying by apoptosis. Climacostol exerts a marked and sustained accumulation of autophagosomes as the result of dysfunctional autophagic degradation. Mechanistic insights showed that climacostol affects autophagosome turnover via p53-AMPK axis, although the mTOR pathway unrelated to p53 levels plays a role. Of note are indications suggesting that climacostol effects on autophagy and apoptosis are actually two separate events, which may act independently on life/death decisions of the cell.

In this picture, the upregulation of the p53 system appears at the molecular crossroads regulating both the anti-autophagic action of climacostol and its role in the induction of apoptosis. 

## 6. The Synthetic Analogues of Climacostol for Biotechnological Applications

The results collected in the last 20 years regarding the activity of climacostol together with the structural properties of other resorcinolic lipids allowed us to design and obtain new synthetic analogues of the protozoan toxin. These compounds shed light on the chemical bases of climacostol actions.

**Figure 5:**
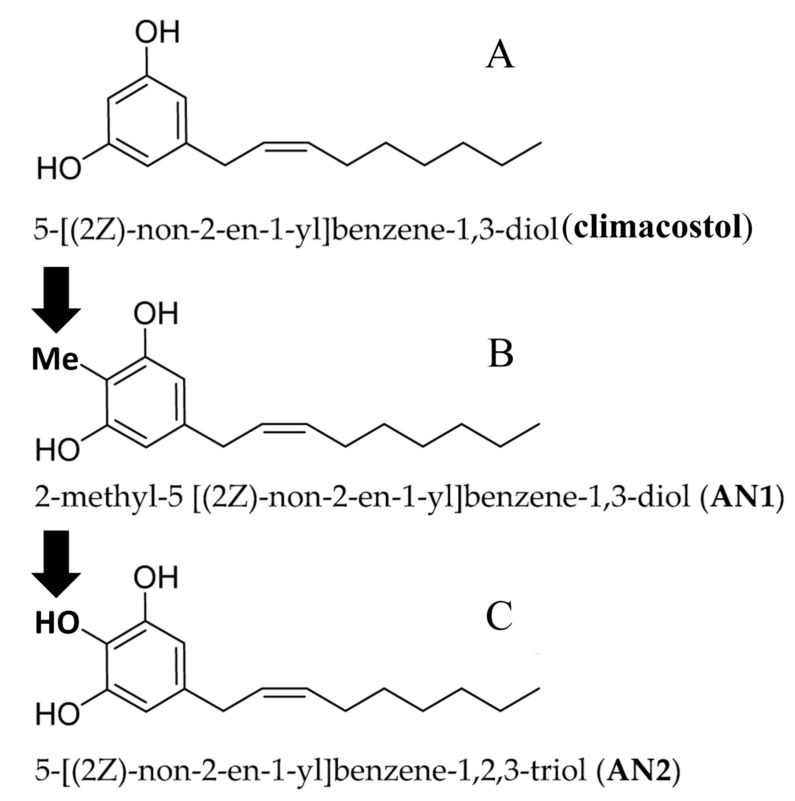
Molecular structures of climacostol and its analogues. Arrows indicate the added groups.

Two analogues, methyl-5 [(2*Z*)-non-2-en-1-yl]benzene-1,3-diol (AN1) and 5-[(2*Z*)-non-2-en-1-yl]benzene-1,2,3-triol (AN2), respectively carrying an additional methyl group and a hydroxyl group in the aromatic ring, were analyzed for their biological activity [31] (Figure 5).

The choice to introduce the aforementioned moieties into the aromatic ring of climacostol was supported by the observation that similar modification performed on some polyphenols and phenolic lipids resulted in a significant improvement of their cytotoxic and antimicrobial activity [37,38].

The effects of AN1 and AN2 were investigated on mammalian cells, pathogenic microbes and free-living ciliated protists, with the main purpose being to identify the structural traits of native climacostol primarily involved in its cytotoxic activities. 

### 6.1. Antitumour Activity

**Figure 6:**
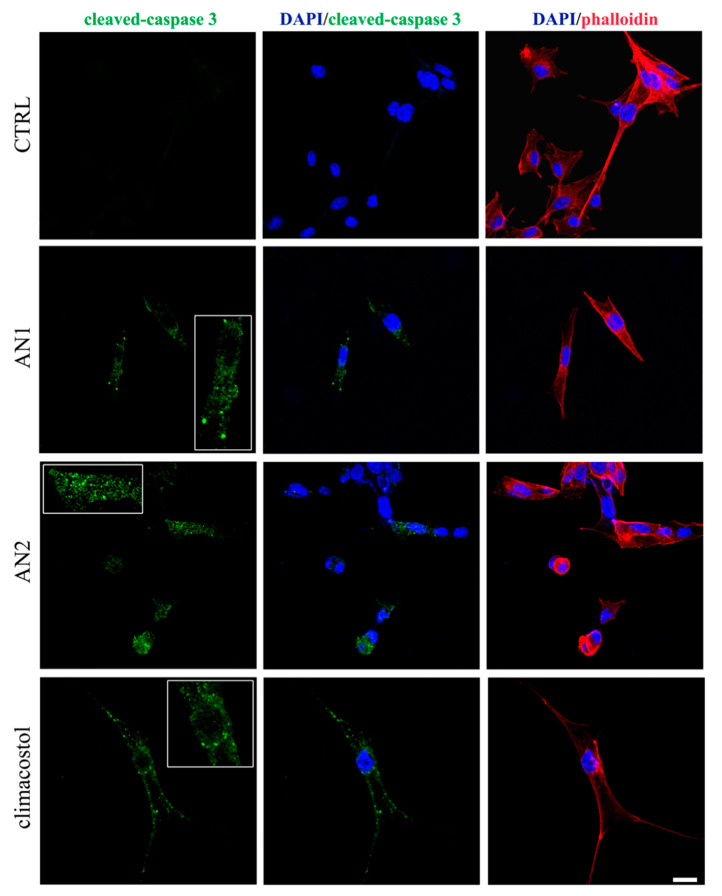
Induction of apoptosis by climacostol and its analogues (AN1, AN2) in melanoma cells. Immunofluorescence of cleaved-caspase 3 (punctate green pattern) in B16-F10 cells cultured in the presence of 30 µg/mL of AN1, AN2, and climacostol or vehicle (CTRL) for 9 h. DAPI (blue) and phalloidin (red) were used for nuclei and cytoskeleton detection, respectively. Inserts represent enlarge image details. Scale bar = 20 µm. Original picture from [31] and reproduced under the Creative Commons attribution 4.0 license: http://creativecommons.org/licenses/by/4.0/.

The pro-apoptotic features of AN1 and AN2 on immortalized cell lines of both tumor (B16-F10, GL261, SK-N-BE, and CT26) and nontumor (C_2_C_12_) origin were analyzed and compared with that of climacostol. The results indicated that cell viability was negatively affected by the two analogues with a comparable strength (Table 1), and both AN1 and AN2 displayed similar or even lower potencies when compared to climacostol [31]. Furthermore, as in the case of various tumor cells exposed to climacostol [4,29,30], immunostaining techniques revealed that B16-F10 melanoma cells expressed high levels of active caspase 3 after AN1 and AN2 treatment (Figure 6), thus demonstrating the activation of an apoptotic pathway induced by both analogues.

### 6.2. Antimicrobial Activity 

AN1 and AN2 were also investigated with dose–response experiments performed to compare their cytotoxic potential against a panel of microorganisms comprising bacterial and fungal pathogens, and freshwater ciliates.

The data collected confirmed that both AN1 and AN2 show an appreciable cytotoxicity on all the microorganisms exposed to different concentrations of the two analogues, with the exception of *Escherichia coli* and *Pseudomonas aeruginosa* that proved to be immune to the toxins. AN1 was the most toxic compound against pathogens and ciliates, whereas AN2 effects were comparable to or worse than climacostol. In particular, AN1 showed MIC and MBC values of 8 μg/mL against the Gram-positive *Staphylococcus aureus* and *Enterococcus faecalis*, respectively, and a value of 4 μg/mL against the fungus *Candida albicans*. In experiments with ciliates, the highest toxicity of AN1 (0.64 μg/mL < LC50 < 2.15 μg/mL) was observed against *B. japonicum*, *P. multimicronucleatum*, *S. ambiguum*, and *S. teres* [31].

**Figure 7:**
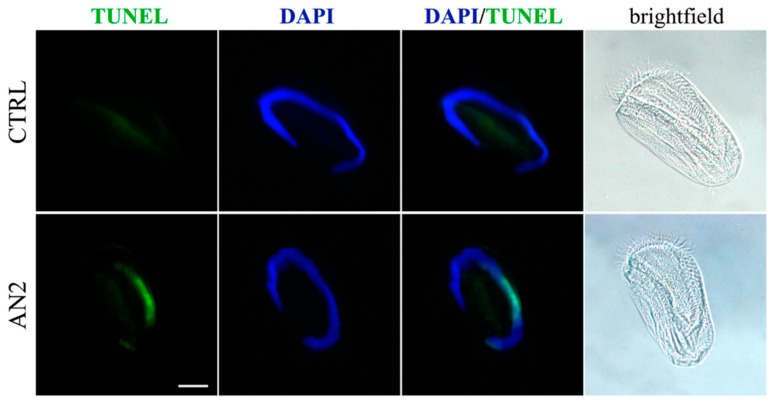
Induction of programmed cell death in ciliated protozoa *Euplotes aediculatus*. TUNEL assay in *E. aediculatus* treated with 2 µg/mL AN2 or vehicle (CTRL) for 2 h. 4′,6-diamidino-2-phenylindole (DAPI) (blue) was used for nuclei detection. Scale bar = 30 µm. Original picture from [31] and reproduced under the Creative Commons attribution 4.0 license: http://creativecommons.org/licenses/by/4.0/.

With regard to the action mechanism of the synthetic toxins, the cytotoxicity of both AN1 and AN2 on some ciliate species appeared to be mediated by a necrotic process, as previously reported for the effect of climacostol on free-living ciliates [17,24]. An exception was represented by the ciliate *Euplotes aediculatus* which displayed the activation of programmed cell death triggered by AN2. In fact, Terminal deoxyribonucleotidyl transferse (TdT)-mediated biotin-16-dUTP Nick-End Labelling (TUNEL) fluorescence assay and light microscopy observations on *Euplotes* specimens exposed to AN2 revealed the typical sequence of events associated with canonical apoptosis, such as the progressive fragmentation of the macronucleus during the early stages in the absence of major changes in cell morphology, and the lack of severe necrotic damage, such as cell swelling and rupture [31] (Figure 7). To summarize, whereas similar effects were substantially observed for climacostol and its analogues on mammalian cells, AN1 was the most active compound against bacterial and fungal pathogens, and protists. The hydroxyl group added to climacostol to obtain AN2 appears to be the pivotal structural trait transforming the protozoan toxin into an apoptosis-inducing compound in unicellular eukaryotes. 

The overall results obtained with AN1 and AN2 encourage the attempt to design and synthetize additional novel analogues of climacostol that can increase or optimize its pharmacological properties.

## 7. Climacostol as a Prodrug

**Figure 8:**
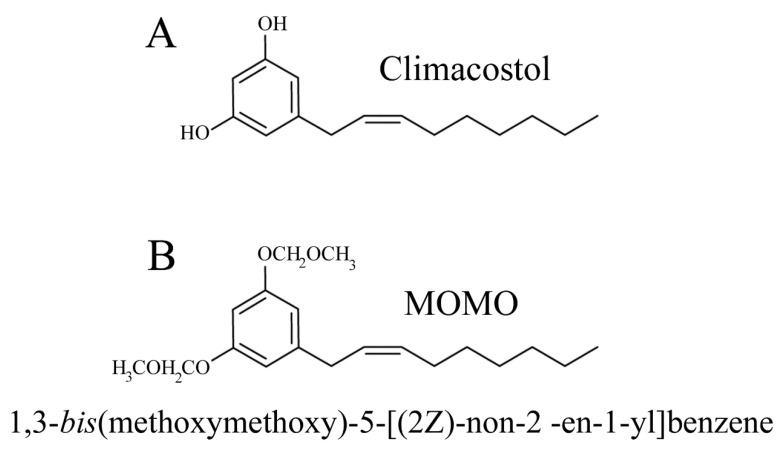
Molecular structures of (**A**) climacostol, and (**B**) MOMO.

During the synthesis of climacostol, the choice of the methoxymethyl ether (MOM) protecting group, which can be removed in a weakly acidic environment, allowed the researchers to efficiently obtain the toxin in the biologically active (*Z*)-configuration climacostol [21] (Figure 8).

This procedure was also applied to design a prodrug strategy based on pH activation, potentially useful to deliver climacostol to acidic pathological multicellular (tumors) or unicellular (parasitophorous of *Leishmania* and digestive vacuole of *Plasmodium*) targets [39]. In fact, the MOM-protected climacostol, which the authors called MOMO, progressively shifts to active climacostol if exposed to pH values lower than 7. This shifting was initially verified in a native biological system (against ciliates other than *C. virens*) where ciliates exposed to MOMO in a physiological culture medium (SMB) at pH = 6.8 did not reveal any sign of toxicity. On the contrary, MOMO resuspended in SMB at pH = 6.3 (not harmful itself for ciliates) induced the same necrotic effect as climacostol [39]. 

**Figure 9:**
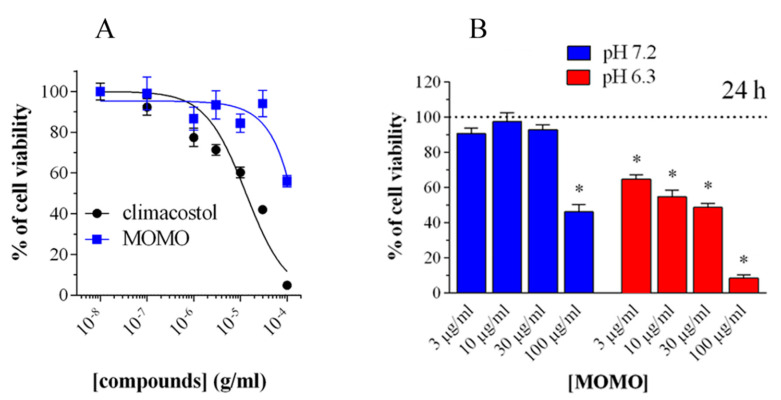
Cytotoxicity of methoxymethyl ether (MOM)-protected climacostol (MOMO) in melanoma cells. (**A**) 3-(4,5-dimethylthiazole-2-yl)-2,5-diphenyltetrazolium bromide (MTT) colorimetric assay on mouse B16-F10 cells, treated with increasing concentrations of MOMO and climacostol for 24 h. (**B**) Increasing concentrations of MOMO in physiologic (pH 7.2) and acidic (pH 6.3) culture medium for 24 h of incubation. Data are representative of at least 6 independent experiments. * *p* < 0.0001 relative to the respective control. Modified from [39] and reproduced under the Creative Commons attribution 4.0 license: http://creativecommons.org/licenses/by/4.0/.

In addition, it was demonstrated that the activity of MOMO could be modulated in a dose-dependent manner to induce cytotoxic or cytostatic effects. The biological activity of MOMO on some tumor cell lines confirmed that this molecule proved toxic in acidic conditions (pH < 6.8), by yielding active climacostol. In these conditions, MOMO induces apoptosis in melanoma cells confirming that it behaves with the same action mechanism as climacostol (Figure 9).

Finally, the safety of MOMO was also assessed in vivo using the fruit fly *Drosophila melanogaster* which is considered a very potent tool to detect the potential damaging effect of new compounds. *Drosophila* flies fed directly with acidic nutrients containing increasing concentrations of MOMO showed a significant reduction in oviposition and survival of larvae, which also exhibited apoptotic cells in the gut, as revealed by TUNEL labeling and cleaved-caspase 3 immunostaining. In addition, mitosis was also reduced in brain tissue of larvae, thus suggesting that active MOMO exhibited prolonged toxic effects after oral intake and gut absorption.

Overall, our data revealed that MOMO efficiently targets different tissues of the developing fly with high metabolic/proliferating activity, such as midgut and brain. 

## 8. Conclusions

Natural products possess enormous structural and chemical diversity that is unsurpassed by any synthetic libraries. Notably, small organic molecules from nature have shown great translational potential [40] since living organisms have a marked ability to synthetize complex molecular structures with defined biological properties, and climacostol is a typical example as a small molecule. However, the possibility to directly purify climacostol as a natural compound from cell cultures is actually limited to very small amounts. Therefore, to address this problem, many efforts have been directed towards the preparation of large amounts of synthetic climacostol and some analogues, through strategies capable of making a cheaper and faster product. 

The studies carried out in recent years on climacostol and its analogues have shown how the alkenylresorcinol structure can exert both appreciable antimicrobial and antitumor activities. The resorcinol moiety is in fact a widespread phenolic unit, which is found in several biologically active natural products [41], as well as a synthetic derivative with biological activities that show considerable therapeutic potential. 

Structure–activity relationship investigations in distinct alkenylresorcinols have shown that an increase in the number of the ring substituents leads to higher cytotoxic activities in many cell lines compared to climacostol. Therefore, it is interesting to note that an increase in the number of hydroxyl groups in climacostol does not lead to an increase in antitumor activity, which can be comparable to or worse than that of the native toxin. In this picture, further synthetic studies to better understand the relationship between the biological activity of climacostol and the position of the substituents in its aromatic ring are currently under development. The biological results will be reported as soon as they are available. 

In conclusion, it appears that small molecule derivatives exhibiting optimum potency/selectivity may have a pivotal role to play in understanding the biochemical machinery underlying cell function/dysfunction as well as in exploiting new therapeutic indications. In this respect, climacostol appears to be a promising lead compound for chemical organic approaches and biotechnological investigations which merit exploitation by drug pharmacology.

## Figures and Tables

**Table 1:** Induction of inhibition of cell viability by means of climacostol and its analogues. EC_50_ (µg/mL) = the concentration producing half of the maximum effect. Original data from [31].

Cell lines	Origin		EC_50_	
		Climacostol	AN1	AN2
B16-F10	mouse melanoma	12.81	18.88	37.94 *
GL261	mouse glioma	28.87	27.32	21.79
SK-NE-BE	human neuroblastoma	19.13	34.31 *	39.84 *
CT26	mouse colon cancer	31.58	36.11	27.42
C_2_C_12_	myoblast of mouse	16.19	29.28 *	31.72 *

* *p* < 0.0001 from climacostol value (F-test); *n* = 3.
